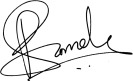# Introduction to special issue: Bioengineered Therapeutics. The Next Generation of Drug Development

**DOI:** 10.1002/btm2.10055

**Published:** 2017-03-31

**Authors:** Pankaj Karande

**Affiliations:** ^1^ Department of Chemical and Biological Engineering,Center for Biotechnology and Interdisciplinary Studies,Rensselaer Polytechnic Institute,Troy, NY 12180

Numerous scientific and technological advances have transformed drug development from an empirical art‐form to a precise and systematic design effort. A fundamental understanding of molecular, cell, and tissue biology has inspired the engineering of a promising new generation of drugs—*Bioengineered Therapeutics*. These comprise native and engineered molecular scaffolds such as proteins, enzymes, and antibodies as drugs, as well as macromolecular assemblies mimicking pathogens and cells as drugs, drug carriers and immunostimulants. Advances in host cell engineering to display molecular libraries of increasingly large and complex diversities; single molecule engineering via site‐directed mutagenesis; structural and biophysical characterization and manipulation of macromolecules and macromolecular architectures; and microfabrication approaches to create drug‐in‐device systems have opened novel avenues for designing bioengineered therapeutics. In parallel, advances in biosynthesis and bioprocessing have provided scalable technologies for manufacturing of bioengineered therapeutics to meet clinical needs.

I am pleased to introduce this special issue of *Bioengineering and Translational Medicine* to our readers. It features an impressive collection of research articles and state‐of‐art reviews, from leaders in academia and industry, from diverse disciplinary backgrounds, that highlight discoveries and innovations in three key areas of bioengineered therapeutics development—(a) Design and discovery approaches, (b) Delivery platforms, and (c) Synthesis and manufacturing technologies.

Rapid discovery and validation of lead candidates is critical to any drug development program for accelerating the clinical entry of most promising therapeutics. This issue features two contributions highlighting novel approaches and platforms to address this need. Cochran and colleagues demonstrate the feasibility of strategically engineering native ligands such as VEGF to create variants that are potent antiangiogenesis agents.^1^ Indeed, the approach of using a natural ligand as a template combined with yeast surface display to rapidly identify potent mutants can be extended to other ligands to develop novel therapeutic antagonists against a broad range of targets. Gorelik and Sidhu offer an informative review on engineered enzyme variants, using ubiquitin as a prototypical example, for discovery of therapeutic candidates.^2^ Beyond design of therapeutics, there is a tremendous potential for employing such schemes to validate and discover new functions for targets, and characterize new modulatory sites.

The drug development endeavor has suffered, disproportionately, from promising leads failing in clinical trials due to poor bioavailability and off‐target effects. Delivery to in situ targets remains a significant challenge. There is a conscious paradigm shift to combine discovery and delivery efforts early in the drug development process to mitigate failure. Seven contributions in this issue discuss novel approaches, both synthetic and biomimetic, to achieve effective delivery by employing discoveries and innovations in biology, immunology, biochemistry, biophysics, and micro‐fabrication.^3^, ^4^, ^5^, ^6^, ^7^, ^8^, ^9^ Champion and colleagues describe the biochemical tailoring of nanoparticles with molecular adjuvants to generate specific effector responses against pathogens that offer superior vaccination against infectious diseases.^3^ Alongside, they present a systematic scheme for engineering physical properties such as shape, size and stiffness of nanoparticle vectors for tuning the Fc‐mediated interactions with macrophages during phagocytosis.^4^ Muro and Manthe describe the design of targeted nanocarriers that sequester overexpressed inflammatory agents in disease pathologies providing an attractive approach for design of new therapeutic systems.^5^ Swartz and colleagues review the potential for use of virus like particles (VLPs) as highly effective delivery vehicles that overcome the many challenges encountered in targeted delivery of therapeutic cargoes.^6^ Desai and colleagues demonstrate inkjet printing of therapeutics in microdevices to circumvent the challenges of low bioavailability, degradation, poor cellular permeability, and low residence time for oral administration.^7^ Goto and colleagues describe a simple and easy to use vaccine administration method using a solid‐in‐oil nanodispersion system incorporating immunomodulatory adjuvants for soliciting specific immune responses via transcutaneous vaccination.^8^ And finally, Rege and colleagues review state‐of‐art approaches in isolation and purification of exosomes, and their potential use in cancer vaccines, drug delivery, and diagnostics. Collectively, these articles present several exciting and innovative approaches for targeted drug delivery.

Although clinical efficacy is a key determinant of the translational potential of a therapeutic, the human impact on patients from translation cannot be achieved unless there is a scalable and robust platform to manufacture the therapeutic in necessary quantities. Industrial processes for biomanufacturing and bioprocessing of therapeutics carry substantially different challenges compared to laboratory or pilot‐scale processes. Two contributions in this issue specifically address these challenges. Shukla and colleagues provide a comprehensive review of antibody manufacturing platforms that have been a key enabler for antibody drug discovery efforts to seamlessly translate into clinical and commercial successes.^10^ Alongside, Linhardt and colleagues offer a complementary and insightful review of challenges and opportunities associated with the manufacturing of polysaccharide therapeutics such as heparin that face orthogonal challenges of a fragile, and on occasion, an unsafe supply chain.^11^ Emerging innovations in chemical synthesis, chemoenzymatic synthesis, and metabolic engineering are poised to offer unique and novel solutions for manufacturing therapeutics of the future.

In summary, this issue provides a broad outlook on the challenges and opportunities for the future of Bioengineered Therapeutics. I am confident that the readership of *Bioengineering and Translational Medicine*, and beyond, will find this issue informative, educational, and thought‐provoking. I am excited about the future of Bioengineered Therapeutics.